# Deep Learning-Based Segmentation and Quantification of Cucumber Powdery Mildew Using Convolutional Neural Network

**DOI:** 10.3389/fpls.2019.00155

**Published:** 2019-02-15

**Authors:** Ke Lin, Liang Gong, Yixiang Huang, Chengliang Liu, Junsong Pan

**Affiliations:** ^1^School of Mechanical Engineering, Shanghai Jiao Tong University, Shanghai, China; ^2^School of Agriculture and Biology, Shanghai Jiao Tong University, Shanghai, China

**Keywords:** powdery mildew, cucumber leaf, convolutional neural network, image segmentation, deep-learning

## Abstract

Powdery mildew is a common disease in plants, and it is also one of the main diseases in the middle and final stages of cucumber (*Cucumis sativus*). Powdery mildew on plant leaves affects the photosynthesis, which may reduce the plant yield. Therefore, it is of great significance to automatically identify powdery mildew. Currently, most image-based models commonly regard the powdery mildew identification problem as a dichotomy case, yielding a true or false classification assertion. However, quantitative assessment of disease resistance traits plays an important role in the screening of breeders for plant varieties. Therefore, there is an urgent need to exploit the extent to which leaves are infected which can be obtained by the area of diseases regions. In order to tackle these challenges, we propose a semantic segmentation model based on convolutional neural networks (CNN) to segment the powdery mildew on cucumber leaf images at pixel level, achieving an average pixel accuracy of 96.08%, intersection over union of 72.11% and Dice accuracy of 83.45% on twenty test samples. This outperforms the existing segmentation methods, K-means, Random forest, and GBDT methods. In conclusion, the proposed model is capable of segmenting the powdery mildew on cucumber leaves at pixel level, which makes a valuable tool for cucumber breeders to assess the severity of powdery mildew.

## Introduction

Powdery mildew is a common fungal disease that mainly infects plant leaves. The hazards of powdery mildew are considerable and may affect photosynthesis (Watanabe et al., 2014). Indeed, when the disease is severe, the infected leaves will shed (Marçais and Desprez-Loustau, 2014), causing significant losses (Xia et al., 2016).

Therefore, it is particularly important to automatically recognize powdery mildew on plant leaves. A number of high-quality image-based methods have been developed to recognize diseases on plants (Mutka and Bart, 2015), including chlorophyll fluorescence imaging, hyperspectral imaging, thermal imaging and visible light imaging. Chlorophyll fluorescence emission, an invisible phenomenon, changes when plants are experiencing biotic and abiotic stresses (Baker, 2008). Thus, chlorophyll fluorescence imaging can be used to measure this trait. Hyperspectral imaging is a technique that can be used to obtain the spectrum for each pixel in the image of a scene, which has been widely used in plant breeding (Dale et al., 2013). In addition, some fungi can affect the transpiration of the leaves and affects the temperature of the surface of the leaves (Lindenthal et al., 2005). Thus, thermal imaging can be employed to measure the temperature of leaves to identify the different types of disease. Methods based on the chlorophyll fluorescence, hyperspectral, and thermal images require expensive equipment and sophisticated analysis methods. In contrast, visible-spectrum RGB images can be obtained using a large number of very accessible devices. As a result, it is possible to gather the data required by more sophisticated algorithms. Therefore, in recent years, many methods for detecting plant diseases using visible-spectrum images have been developed.

Based on the Hough transform of the image and the random forest algorithm, Wspanialy and Moussa (2016) built a detection machine vision system to detect early powdery mildew. In the field testing on a greenhouse of tomato plants, this method achieved 85% recognition accuracy. Zhang et al. (2017) had combined the shape and color features from the disease regions and used sparse representation classification to recognize diseased leaf images. The method they proposed was feasible in recognizing seven major diseases of cucumber, and it achieved 85.7% recognition accuracy in their test datasets. With the development of deep leaning in computer vision tasks, especially convolutional neural networks (CNN), researchers can achieve higher recognition accuracy in object detection and semantic segmentation tasks. Therefore, deep learning might be used in automatic plant disease identification (Barbedo, 2016). At present, there have been many studies using CNN for plant disease recognition. A plant disease classification model was developed by Sladojevic et al. (2016), which could distinguish 13 different types of plants disease including powdery mildew from the images of healthy plant leaves. Another study using CNN to classify diseases of plants was (Amara et al., 2017). They used the *LeNet* architecture to classify banana leaf diseases. In order to overcome the problem of the slow recognition speed of neural networks, Fuentes et al. (2017) proposed a real-time tomato plant disease and pests recognition model, which could recognize nine diseases including powdery mildew. There are also a number of studies using CNN to classify plant diseases, including (Mohanty et al., 2016; Wang et al., 2017; Ferentinos, 2018).

Notably, current image-based models commonly regard the powdery mildew identification problem as a dichotomy case, yielding a true or false classification assertion. However, quantitative evaluation of the disease resistance traits plays an important role in plant variety screening for breeders. Thus, there is an urgent need to exploit the extent to which the leaves are infected.

In this paper, we proposed a new deep learning scheme which represents powdery mildew infection by masked regions generated from the segmentation model. In this way, the exact severity of the disease can be obtained. Compared to the hyperspectral image-based method, the proposed method is easier to implement and does not require expensive special imaging equipment. Further, compared to methods based on visible image classification, our method is able to obtain the location of the disease regions. With this advantage, the proposed method can provide the area and shape of the disease regions. The former can be used to indicate the severity of the disease, and the latter can help with the morphological analysis of the disease regions. Our method is available under the open-source MIT License at *https://github.com/ChrisLinSJTU/segmentation-of-powdery-mildew*.

K-means is a typical unsupervised method that can be used for clustering. Zhang et al. (2017) employed K-means method to segment the disease regions in plant leaves. While, Random forest and Gradient boosting decision tree (Ke et al., 2017) are supervised learning methods that can be used to deal with classification and regression problems. Therefore, these three methods can be applied to classify the pixels in an image to segment the disease region. Consequently, we compared the proposed method to these three segmentation methods. However, compared with the deep learning-based methods, these three methods have lower model complexity, which means that the representation ability of these three methods is not as powerful as deep learning-based methods. Experimental results also showed that our method is superior to these three methods.

The rest of this paper is organized as Materials and Methods followed by Results and Discussion. In the Materials and Methods section, we collected image samples and proposed a convolutional neural network based on U-net. Fifty cucumber leaves infected with powdery mildew were collected, and the annotations of all cucumber leaf images were manually created. Thirty pairs (images and annotations) of them were used for training and twenty pairs were used for testing. Image augmentation techniques are used for better training the sematic segmentation model. To obtain a more robust model, we used a custom loss function and added a batch normalization (Ioffe and Szegedy, 2015) layer behind each convolutional layer. In the Result section, we used six metrics, including pixel accuracy, intersection over union (Long et al., 2015), Dice accuracy (Milletari et al., 2016), Recall, Precision and F_β_ score to show the results of the proposed model on twenty test samples. In addition, we compared these six metrics with the existing K-means, Random forest, and GBDT image segmentation methods. In the Discussion section, we discussed the importance of the proposed model and some findings in the experimental results.

## Materials and Methods

The image acquisition process is demonstrated in section “Sample Collection,” and in section “Image Preprocessing, Network Structure of the Image Segmentation Model, Network Training, and Model Testing” we describe the pipeline of our method.

### Sample Collection

**FIGURE 1:**
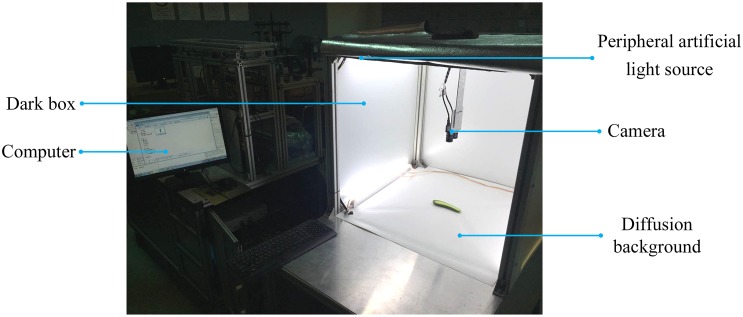
*In vitro* Cucumber Fruit/Leaf Phenotyping platform.

**FIGURE 2:**
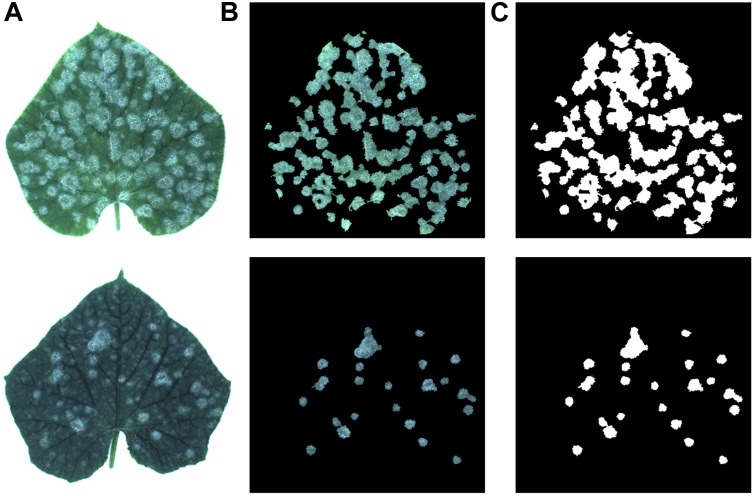
**(A)** Two samples of cucumber leaves, **(B)** their disease areas, **(C)** annotation of infected areas.

In this paper, 50 cucumber leaves infected with powdery mildew were collected from Shanghai, China. The images of these samples were captured in a Cucumber Fruit Leaf Phenotype Automated Analysis Platform. It is an image-based cucumber phenotype platform whose shape is an 80 cm × 80 cm × 140 cm rectangle. A USB camera with a resolution of 2592 × 1944 × 3 is on top of it for photographing plants. There is a diffusion background at the bottom for providing uniform illumination and a peripheral artificial light source at the top for minimizing the shadow. In addition, there is a computer next to sample holding area that is used to perform phenotypic analysis. The platform is shown in Figure 1. Figure 2A shows two samples of cucumber leaves infected with powdery mildew.

To train the CNN for identifying disease areas on the leaves, it is necessary to annotate the ground truth. Therefore, the annotations of all the cucumber leaf images were manually created. Figure 2B shows the disease areas of the cucumber leaves. Figure 2C shows the annotation images of each sample, in which the pixels of disease regions were annotated as white and the rest were annotated as black.

In these 50 images and their annotations, we randomly selected 30 pairs (images and its annotations) as a training set to train our convolutional neural network and 20 pairs as a test set to evaluate the performance of the algorithm.

### Image Preprocessing

The background of the samples we collected was white, while the main feature in the powdery mildew regions is also white. Thus, it might be difficult to achieve good performance by directly using the samples with white background for training. Consequently, it is necessary to adjust the background color to black. The process of separating a leaf from image was performed with following steps: (1) an image was transformed into the HSV color space, (2) the S channel was extracted and the OTSU method (Otsu, 1979) was applied to it to obtain the mask, and (3) the RGB channels of the original picture were multiplied by the mask to obtain a picture with a black background. In addition, the images were downscaled to 512 × 512 × 3 by down-sampling.

### Network Structure of the Image Segmentation Model

**FIGURE 3:**
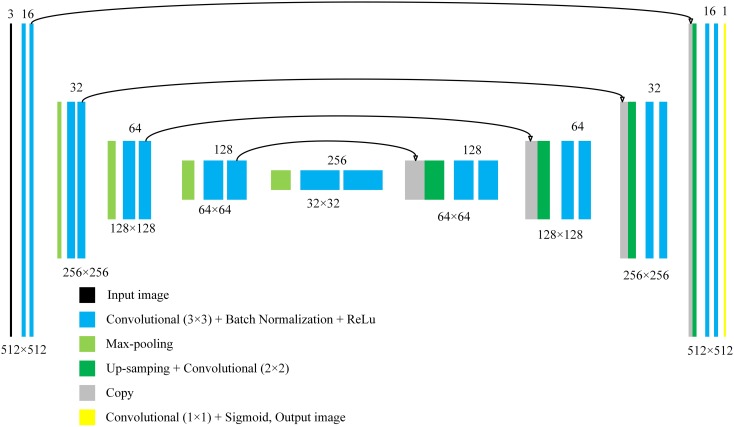
The structure of the proposed model.

The convolutional neural network constructed in this paper is mainly based on the U-Net. U-net is one of the convolution neural networks that had shown excellent performance in biomedical image segmentation (Ronneberger et al., 2015). It is characterized by the Up-sampling layer and the concatenation of the Up-sampling layer and the previous activation layer. The process of Up-sampling makes the output of the neural network the same size as the input image, achieving pixel-level segmentation. In addition, the process of concatenation enables precise positioning of the target. These two processes are very appropriate for pixel-level segmentation of powdery mildew. Moreover, based on massive data augmentation, the network can be trained end-to-end (input is an image, and output is also an image) from very few images. This is very suitable for the agricultural field because, under normal circumstances, there are no large data sets for researcher to train neural networks, especially in the field of phenotypes. The structure of the U-net we constructed in the paper is shown in Figure 3.

In Figure 3, each color block represents a module of the neural network. The number below each color block, such as 512 × 512, represents the size of the output image of the layer. The number above each color block represents the “depth” of the current layer. In the U-net we used, the input is a color image, and the output is a grayscale image. For an output, when the pixel value is greater than 0.5, it is marked as a pixel in a disease area. Compared with the original U-net, we had added a batch normalization layer behind each convolution layers with a 3 × 3 convolution kernel. The addition of batch normalization allows us to use higher learning rates to accelerate the training process, and it also has the effect of regularization (Ioffe and Szegedy, 2015). In addition, after adding the batch normalization layer, the neural network becomes insensitive to weight initialization.

The segmentation of disease regions is essentially a binary classification problem which is performed on each pixel. However, the number of pixels of disease regions are smaller than non-disease regions. Thus, this creates a situation that the positive and negative samples are not balanced, which could make the neural network tend to have a low accuracy on the category with fewer samples (Huang et al., 2016). This could lead to a lower recognition accuracy in disease regions. To solve this problem, based on the binary cross entropy loss function (Goodfellow et al., 2016), we had magnified the loss value of the positive pixels by 10 times, in which the value of 10 was determined empirically. The loss function we used is shown in Eq. 1.

(1) $$L=\overset{m}{\underset{i=1}{\Sigma }}-\left(10\times y_{i}\times \log({y^{\prime }}_{i})+(1-y_{i})\times \log\left(1-{y^{\prime }}_{i}\right)\right)$$

*m* denotes the number of pixels in an image. *y_i_* denotes the real value of the *i-*th pixel, whose value is 0 or 1. *y_i_′* denotes the predicted value of the *i*-th pixel by the method, whose range is 0 to 1.

### Network Training

**FIGURE 4:**
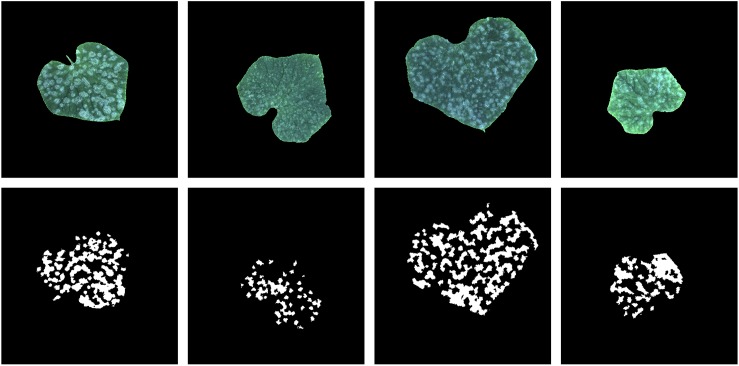
Image augmentation of four samples (images and their annotation).

Since the training sample has only 30 images, we had to expand these 30 images to train the neural network more effectively. Expansion methods include rotation, horizontal and vertical shift, zooming in and zooming out, horizontal flipping and vertical flipping. The range of rotation is 0 to 180 degrees, and the range of horizontal and vertical shift is 0.1 times width and height of the image, respectively; the zoom range is 0.6 to 1.4. The values of the four transformations to an image are all randomly selected from their range. Moreover, when an image was transformed, its annotation image was also transformed in the same way. In addition, since the parameters of the transformations are randomly selected, it is necessary to generate a random number. To ensure the generated data is the same in each epoch during the training process, we fixed the value of the random seed as 1. Based on 30 training samples and transformation methods, 10,000 training data pairs were generated. Four generated images and the correspond annotation images are show in Figure 4.

In the optimization process, the Adam method was applied with the learning rate of 0.0001, and other parameters are consistent with those in the original manuscript (Kingma and Ba, 2014). As for the initialization of the weights, we used the Glorot initialization method (Glorot and Bengio, 2010). We trained our model with the generated 10,000 pairs, where the batch size for each iteration was 2 with 32 epochs.

The hardware used for training the model is a GPU server equipped with an Intel Xeon E5-2620 CPU and an NVIDIA TESLA P100 GPU. We implemented our model with a high-level neural network API called Keras (Chollet, 2015) with the Tensorflow (Abadi et al., 2016) backend running on the Ubuntu 16.04 operating system.

### Model Testing

Pixel-level segmentation of images is also known as semantic segmentation, in which the common metrics include pixel accuracy, intersection over union (Long et al., 2015) and dice accuracy (Milletari et al., 2016). The equations of these three metrics are shown in Eqs 2, 3, and 4, where *p_tf_* denotes the number of pixels which are marked as disease regions by both the output of the algorithm and the ground truth in an image; *p_t_* and *p_f_* denote the number of pixels which are marked as disease regions by the ground truth and the output of the algorithm, respectively. In this paper, we use these three metrics to assess the performance of the method.

(2) $${Acc}_{Pixel}=\frac{1}{m}\overset{m}{\underset{i=1}{\Sigma }}f_{i},f_{i}=\left\{ \begin{array}{c} 1\,\;y_{i}={y^{\prime }}_{i}\\ 0\,\;y_{i}\neq {y^{\prime }}_{i} \end{array}\right.$$

(3) $${Acc}_{IU}=\frac{p_{tf}}{p_{t}+p_{f}-p_{tf}}$$

(4) $${Acc}_{Dice}=\frac{2\times p_{tf}}{p_{t}+p_{f}}$$

To verify the performance of the proposed model, we used 20 samples to test it. The three metrics mentioned above, IU accuracy, Dice accuracy and Pixel accuracy, were used to evaluate the performance of the model. Since the final output of our model is a 512 × 512 grayscale image and the values of all pixels vary from 0 to 1, a threshold, whose value is 0.5, was set to binarize the output to obtain the segmented region. Recall, Precision and F_β_ (Powers, 2011) were also used to evaluate the performance of the model. Generally, for disease recognition, all disease areas are supposed to be detected by the algorithm. As a consequence, Recall usually has priority over Precision. So, we set the β in F_β_ as 2, which means the Recall is twice as important as the Precision.

Zhang et al. (2017) applied a sparse representation classification method to recognize multiple diseases on cucumber leaves, in which the K-means method was employed to segment the disease regions. Therefore, we also compared our model with the K-means disease segmentation method in detail.

## Results

### Results of 20 Test Samples

**FIGURE 5:**
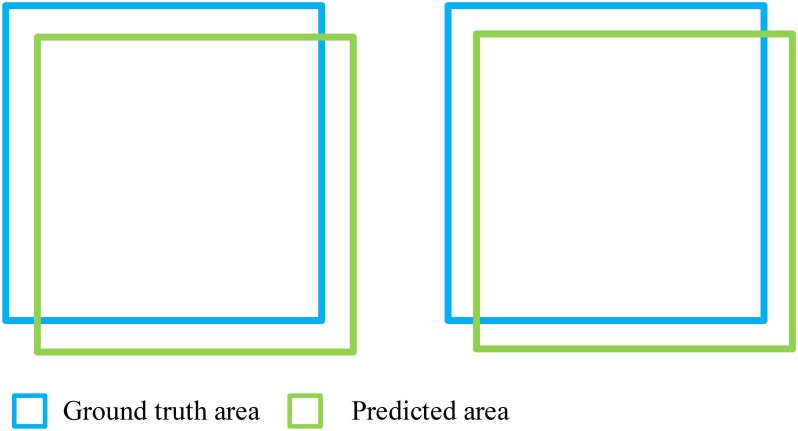
Situation when Dice acc and IU acc are 0.8 (left) and 0.7 (right).

Our model achieved satisfactory segmentation accuracy on 20 test samples. The result of IU accuracy, Dice accuracy and Pixel accuracy of the proposed model and the K-means method are shown in Table 1. Our models performed better than the K-means method on these three metrics. The average IU, Dice and Pixel accuracy of the former are 72.11, 83.45, and 96.08%, respectively, while the latter are 47.05, 63.11, and 92.33%, respectively. Generally, in the same segmentation performance, the value of Dice accuracy is usually greater than IU accuracy. For Dice accuracy, 0.8 can be a good value, while 0.7 is good for IU accuracy. Figure 5 shows the situation when Dice accuracy and IU accuracy are 0.8 and 0.7, respectively, in which they almost have the same segmentation performance.

**Table 1:** Accuracy of our model and K-means method in 20 test samples^∗^.

No.	Our model IU acc.	Our model Dice acc.	Our model Pixel acc.	K-means IU acc.	K-means Dice acc.	K-means Pixel acc.
1	69.93%	82.31%	97.76%	36.07%	53.02%	93.55%
2	81.92%	90.06%	95.65%	46.96%	63.91%	89.17%
3	53.98%	70.12%	99.24%	14.55%	25.41%	96.64%
4	83.41%	90.95%	94.73%	44.89%	61.96%	84.93%
5	82.35%	90.32%	96.88%	66.46%	79.85%	94.75%
6	73.04%	84.42%	96.17%	57.79%	73.25%	94.73%
7	82.68%	90.52%	95.78%	62.88%	77.21%	92.01%
8	83.11%	90.77%	95.60%	49.55%	66.26%	88.34%
9	63.33%	77.55%	96.79%	40.80%	57.95%	95.61%
10	71.71%	83.53%	96.67%	51.00%	67.55%	94.79%
11	73.00%	84.40%	96.43%	58.14%	73.53%	95.24%
12	79.20%	88.39%	96.98%	59.01%	74.22%	94.50%
13	64.31%	78.28%	97.76%	39.77%	56.91%	95.72%
14	85.65%	92.27%	94.42%	45.33%	62.38%	81.33%
15	65.78%	79.36%	93.18%	45.82%	62.84%	90.47%
16	67.21%	80.39%	95.14%	46.27%	63.27%	92.79%
17	54.09%	70.20%	95.34%	32.46%	49.01%	91.85%
18	72.71%	84.20%	93.90%	51.34%	67.85%	91.20%
19	64.99%	78.78%	96.33%	49.41%	66.14%	95.27%
20	69.76%	82.19%	96.80%	42.51%	59.65%	93.76%


*^∗^The average accuracy values of IU, Dice, Pixel of the proposed model in test samples are 72.11, 83.45, and 96.08%, respectively; the average accuracy values of IU, Dice, Pixel of the K-means method in test samples are 47.05, 63.11, and 92.33%, respectively.*

The results of Precision, Recall and F_2_-score of our model and K-means method are shown in Table 2. The average Precision, Recall and F_2_-score of the former are 73.30, 97.34, and 91.20%, respectively, while the latter are 71.35, 60.55, and 60.83%, respectively. The precision of the proposed model is not very good, but the recall is quite high, which means the model has a certain degree of over-segmentation. A further explanation is that the most disease regions had been recognized; however, some non-disease areas had been misidentified as disease areas. This situation is acceptable, because, for disease detection, the disease regions are not supposed to be missed by the algorithm.

**Table 2:** Precision, Recall and F-score of our model and K-means method^∗^.

No.	Our model Precision	Our model Recall	Our model F2 Score	K-means Precision	K-means Recall	K-means F2 Score
1	70.69%	98.48%	91.30%	43.08%	68.92%	61.54%
2	82.10%	99.74%	95.63%	93.61%	48.52%	53.69%
3	56.90%	91.32%	81.46%	16.20%	58.86%	38.56%
4	83.57%	99.77%	96.04%	94.00%	46.21%	51.44%
5	82.80%	99.34%	95.52%	91.17%	71.04%	74.32%
6	73.86%	98.50%	92.34%	78.70%	68.51%	70.33%
7	83.10%	99.39%	95.64%	91.50%	66.78%	70.60%
8	83.55%	99.37%	95.74%	89.44%	52.63%	57.35%
9	64.47%	97.30%	88.31%	63.78%	53.10%	54.94%
10	72.42%	98.66%	91.99%	72.42%	63.30%	64.93%
11	73.32%	99.42%	92.81%	79.89%	68.11%	70.18%
12	79.50%	99.53%	94.76%	81.04%	68.47%	70.66%
13	66.39%	95.35%	87.70%	49.58%	66.79%	62.45%
14	85.88%	99.68%	96.58%	95.43%	46.34%	51.65%
15	71.35%	89.38%	85.08%	73.48%	54.89%	57.82%
16	68.33%	97.63%	89.91%	65.84%	60.89%	61.82%
17	59.08%	86.48%	79.14%	40.65%	61.70%	55.90%
18	73.13%	99.22%	92.61%	84.52%	56.67%	60.67%
19	65.46%	98.89%	89.72%	65.23%	67.06%	66.69%
20	70.04%	99.44%	91.74%	57.37%	62.13%	61.12%


*^∗^The average Precision, Recall and F2 score of our model in test samples are 73.30, 97.34, and 91.20%, respectively; the average Precision, Recall and F2 score of K-means method in test samples are 71.35, 60.55, and 60.83%, respectively.*

In addition, we also compared the proposed method to the Random forest method and GBDT (Ke et al., 2017) method. Although these two methods are supervised learning method, usually used for classification and regression, they also can be used to image segmentation regarding pixels as classification targets. As above, 30 images were used for training and 20 images were used for testing. Each image contains 262,144 pixels (512 × 512), so the training set contains a total of 7,861,320 samples. Testing set contains 5242,880 samples. Lightgbm (Ke et al., 2017) and scikit-learn (Pedregosa et al., 2011), two Python packages, were used to implement these two methods separately. The results show that the proposed methods have the best performance in terms of IU accuracy, Dice accuracy, Pixel accuracy, and Recall in twenty test images. However, for the metric of Precision, the average accuracy of our method is slightly lower than GBDT. These can be seen in Table 3.

**Table 3:** The performance of our method and the three other methods^∗^.

Method	Precision	Recall	F2 score	IU acc.	Dice acc.	Pixel acc.
The proposed method	73.30%	97.34%	91.20%	72.11%	83.45%	96.08%
GBDT	73.90%	70.81%	70.86%	56.96%	71.44%	94.33%
Random Forest	70.99%	69.33%	69.20%	54.84%	69.46%	93.95%
K-means	71.35%	60.55%	60.83%	47.05%	63.11%	92.33%


*^∗^The average accuracy of Precision, Recall, F2 score, IU accuracy, Dice accuracy, and Pixel accuracy of the proposed model and three other methods.*

### Output of 3 Samples by Proposed Model

**FIGURE 6:**
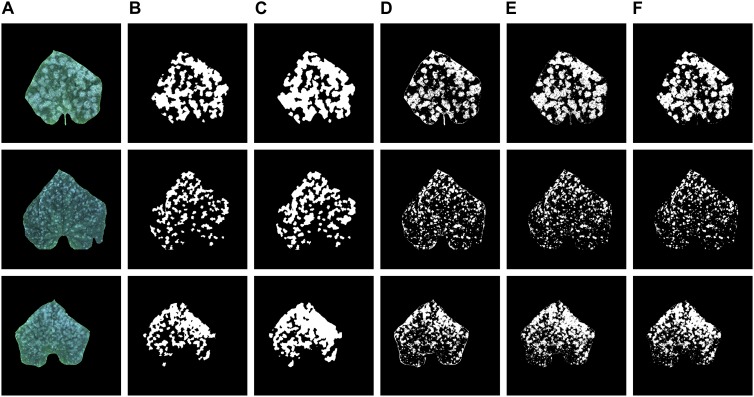
**(A)** Original images, **(B)** annotation images, **(C–F)** recognition results of the proposed model, K-means, Random forest, and GBDT methods.

Figure 6 shows the recognition results of the proposed model, K-means, Random forest, and GBDT methods on three test samples, which include the original images, the annotation image, the segmentation results of the proposed model and the segmentation results of the other three methods. As can be seen in Figure 6, when compared to the annotation images, the prediction results of the proposed model have greater predicted areas, which is consistent with the relatively high Recall.

As for the prediction result of the K-means, Random forest, and GBDT methods, the areas of the segmentation are relatively small. Thus, it leads to a unilateral bias of under segmentation of the infected disease regions, which is evident in Figure 6D–F.

### Visualization of the Feature Map of CNN Model

**FIGURE 7:**
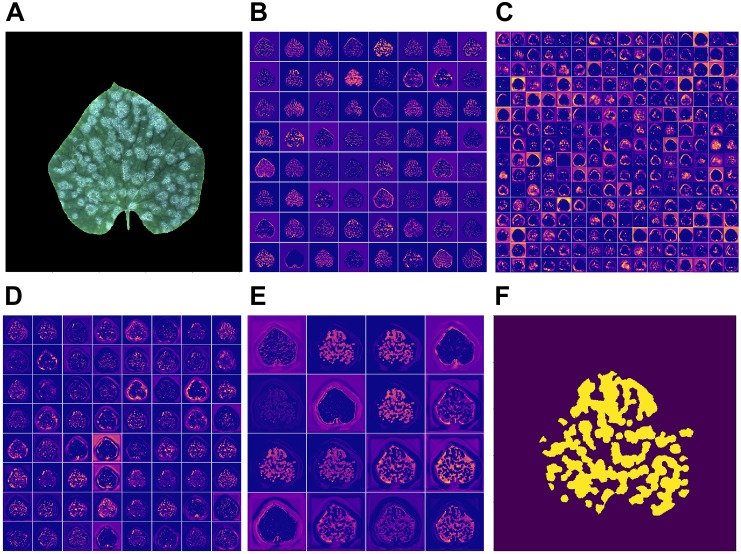
**(A)** Input image; **(B–E)** feature map of the proposed model given this input image; **(F)** output image.

Feature map opens the gray box of a deep-learning based model, illustrating the intermediate result of the learning process. Figure 7 shows the feature map of the middle layers and an output image (f) produced by the network when given the input image (a). Figure 7B–D show the output of the activation layer after the sixth, tenth, and fourteenth convolutional layers, respectively. Figure 7E shows the output of the last activation layer.

**FIGURE 8:**
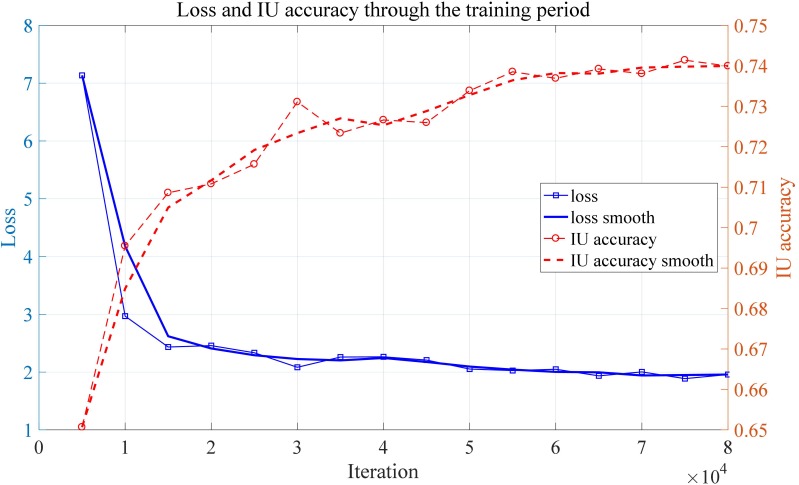
Loss and IU accuracy through the training period.

As can be seen from the Figure 7B, the edge of the leaf the disease regions are highlighted by the convolutional neural network. When it comes to the output of the middle layer which is shown in Figure 7C, the feature map appears to be more abstract. This is because the middle layer of a neural network is difficult to interpret in general. In Figure 7D, the output of the convolutional neural network has no obvious sharp edges. The edges of the leaf gradually fade, which is expected because the model is supposed to pay more attention to the disease region rather the edge of a leaf. In the output of the activation layer shown in Figure 7E, which is close to the output layer, the disease region becomes more concentrated. Figure 8 shows the convergence process of the loss function value and IU accuracy of the proposed segmentation model during the period of training, in which the bold line is the result of smoothing the original curve for better demonstration.

## Discussion

This study aimed to tackle the problem of segmenting powdery mildew on leaves accurately based on visible images. To address this problem, we proposed a convolutional neural network model based on the U-net architecture which is used for sematic segmentation tasks in the field of computer vision. Experimental results on 20 test samples demonstrated that, compared to the existing K-means, Random forest, and GBDT image segmentation methods, the proposed method greatly improved the accuracy of powdery mildew segmentation. However, the proposed method may have greater computational complexity, which means it might be hard to deploy the proposed method to portable device.

Compared to some feature-based plant disease identification methods, this method alleviates researchers from manually extracting complex features in the image and designing complicated analytical methods. In addition, compared with some existing methods based on deep learning for classifying and identifying disease on plant leaves, our method can segment powdery mildew on a cucumber leaf at the pixel level. In summary, the principal discoveries include:

1. In twenty test samples, our model achieved a satisfactory segmentation accuracy of powdery mildew under three metrics of IU accuracy, Dice accuracy and Pixel accuracy. Moreover, the Pixel accuracy of all samples is relatively high, which means that the performance of the proposed model when segmenting powdery mildew on cucumber leaves is feasible in practice. We also randomly selected three samples from twenty test samples to compare the output of the proposed model and the three other methods. The mask image output by the proposed model had a certain degree of over-segmentation when segmenting powdery mildew. However, the mask image obtained by the K-means method had a certain degree of under-segmentation. In addition, the edges of the predicted area of the proposed model were smoother than the K-means method. Generally speaking, the regions of powdery mildew usually appear in block form. Therefore, the smoother edge of the disease region is expected.
2. Unbalanced positive and negative samples in the image cause relatively high segmentation accuracy, in which there are more pixels belonging to the background. Furthermore, the background might be easier to be recognized than the foreground.

In addition, we also found an interesting phenomenon where, in some test samples (such as sample number 3), the Pixel accuracy is high, while the IU accuracy and Dice accuracy are relatively low. After analyzing the image of this sample and the output mask of the K-means algorithms, we found that the area of the disease region in the image was very small. Since the non-disease area is easier to identify, the Pixel accuracy is very high in sample number 3. On the metric of Recall, our model achieved good accuracy on these twenty samples. In general, Recall and Precision are a pair of contradictory metrics. Higher Recall typically corresponds to lower Precision, which explains the paradox of segmentation of powdery mildew in cucumber leaves. In general, higher Recall is preferred because it can lead to the production of models which miss less disease regions. Analysis and experimentation reveal that the proposed convolutional neural network based on the U-net can segment powdery mildew on cucumber leaves accurately at pixel level and can improve on the segmentation accuracy of the existing methods. The improvement of segmentation accuracy helps to estimate the severity of powdery mildew on leaves more accurately, which makes our improved software a valuable tool for cucumber breeders.

However, it is worth noting that there are some limitations in this method. Given the fact that the images are collected on our platform, to implement the proposed method, the images need to be captured under controlled conditions, not in the field. In addition, the insufficient size and variety of annotated datasets, in which symptoms caused by other disorders are not contained in our dataset, may be a factor that influences the performance of deep learning methods (Barbedo, 2018). Thus, other types of leaf damage should be minimal absent.

## Author Contributions

KL, LG, and YH conducted mathematical modeling and article writing. KL also completed the software development and experimental verification. CL and JP supervised the whole project and conducted the experimental verification.

## Conflict of Interest Statement

The authors declare that the research was conducted in the absence of any commercial or financial relationships that could be construed as a potential conflict of interest.
